# Using learning analytics in clinical competency committees: Increasing the impact of competency-based medical education

**DOI:** 10.1080/10872981.2023.2178913

**Published:** 2023-02-23

**Authors:** Patricia A. Carney, Stefanie S. Sebok-Syer, Martin V. Pusic, Colleen C. Gillespie, Marjorie Westervelt, Mary Ellen J. Goldhamer

**Affiliations:** a Professor of Family Medicine, Oregon Health & Science University, Portland, OR, USA; b Emergency Medicine, Stanford University School of Medicine, Palo Alto, CA, USA; c Emergency Medicine, Harvard Medical School, Boston, MA, USA; d Medicine, New York University New York, NY, USA; e Director of Assessment, Evaluation and Scholarship, University of California, Davis, CA, USA; f Medicine, Harvard Medical School, Massachusetts General Hospital, and Mass General Brigham, Boston, MA, USA

**Keywords:** Learning Analytics, Competency-based medical education, graduate medical education, Clinical Competency Committees, Ressident Assessment

## Abstract

Graduate medical education (GME) and Clinical Competency Committees (CCC) have been evolving to monitor trainee progression using competency-based medical education principles and outcomes, though evidence suggests CCCs fall short of this goal. Challenges include that evaluation data are often incomplete, insufficient, poorly aligned with performance, conflicting or of unknown quality, and CCCs struggle to organize, analyze, visualize, and integrate data elements across sources, collection methods, contexts, and time-periods, which makes advancement decisions difficult. Learning analytics have significant potential to improve competence committee decision making, yet their use is not yet commonplace. Learning analytics (LA) is the interpretation of multiple data sources gathered on trainees to assess academic progress, predict future performance, and identify potential issues to be addressed with feedback and individualized learning plans. What distinguishes LA from other educational approaches is systematic data collection and advanced digital interpretation and visualization to inform educational systems. These data are necessary to: 1) fully understand educational contexts and guide improvements; 2) advance proficiency among stakeholders to make ethical and accurate summative decisions; and 3) clearly communicate methods, findings, and actionable recommendations for a range of educational stakeholders. The ACGME released the third edition CCC Guidebook for Programs in 2020 and the 2021 Milestones 2.0 supplement of the Journal of Graduate Medical Education (JGME Supplement) presented important papers that describe evaluation and implementation features of effective CCCs. Principles of LA underpin national GME outcomes data and training across specialties; however, little guidance currently exists on how GME programs can use LA to improve the CCC process. Here we outline recommendations for implementing learning analytics for supporting decision making on trainee progress in two areas: 1) Data Quality and Decision Making, and 2) Educator Development.

## Introduction

Graduate medical education (GME) and Clinical Competency Committees (CCCs) are evolving to monitor trainee progression using competency-based medical education (CBME) principles and outcomes [1–4]. CBME promotes effective, individualized development of residents [3,5], though evidence suggests CCCs fall short of this goal [6–8], despite CBME advancing for over 20 years [9,10]. Challenges include that evaluation data are often incomplete, insufficient, poorly aligned with performance, conflicting or of unknown quality [11]. CCCs struggle to organize, analyze, visualize, and integrate data elements across sources, collection methods, contexts, and time-periods, which makes advancement decisions difficult [11,12]. These issues are even more urgent in CBME, where *more* data are required for *every* learner to individualize development [13–16]. ‘Learning analytics have significant potential to improve competence committee decision making, yet their use is not yet commonplace [15,16].

Learning analytics is the interpretation of multiple data sources gathered on trainees to assess academic progress, predict future performance, and identify potential issues to be addressed with feedback and individualized learning plans (ILPs) [15,17,18]. What distinguishes LA from other educational approaches is systematic data collection and advanced digital interpretation and visualization to inform educational systems [19,20]. These data are necessary to: 1) fully understand educational contexts and guide improvements; 2) advance proficiency among stakeholders to make ethical and accurate summative decisions; and 3) clearly communicate methods, findings, and actionable recommendations for a range of educational stakeholders [15,16,21].

The ACGME released the third edition CCC ‘Guidebook’ for Programs in 2020 [1] and the 2021 Milestones 2.0 supplement of the Journal of Graduate Medical Education (JGME Supplement) presented important papers that describe evaluation and implementation features of effective CCCs [22]. Principles of LA underpin national GME outcomes data [23] and training across specialties [21,24]; however, our careful review of these documents [1,22] indicate that little guidance currently exists on how GME programs can use LA to improve the CCC process.

In this paper, we outline recommendations for implementing learning analytics for supporting decision making on trainee progress in two areas: (1) Data Quality and Decision-Making and (2) Educator Development.

## Data quality and decision making

We outline our long- and short-term recommendations for data quality and decision making in Table 1. In the near future, data quality standards that are actually embedded in information systems would help GME programs ensure that data quality checks occur *prior to* CCC meetings. In addition, active engagement of residents in the evaluation process [25]; and monitoring of assessor quality are needed [26]. Aspirational goals include implementing systemized data processing to reduce human error [16] and automating ACGME cloud-based data visualization processes to reduce workload redundancies and improve efficiencies.

**Table 1.** Effective Use of Learning Analytics by CCCs: Data Quality and Use in Progression Decision Making.

Core Learning Analytics Components	*Recommendations for More Effective Use of Learning Analytics in CCCs*	
	Practical and Short-Term	Aspirational and Future-Oriented
Data Quantity & Quality	Add data quality standards to determine targets for data readiness. Use process maps and data quality checks to identify systematic missingness and data capture/processing issues. Implement data quality reviews *prior* to CCC meetings to determine which data are ready for decision-making. Engage residents in the data review process [25] Monitor and improve assessor quality [26] Implement processes to continuously monitor and improve data quality (CQI).	Implement systematized data processing to reduce human error. Advance assessments of reliability and validity.
Data Integration & Synthesis	Enhance relationships among stakeholders (e.g., CCC members, program faculty, trainees, hospital leadership and data team) to ensure robust data capture Fully integrate data team as active CCC participants to assist with display, synthesis, and interpretation of multi-source evaluations [27] Establish processes to determine if intervention is needed at the program or trainee level Implement data sharing processes with trainees for co-production of ILPs [28–30]	Collaborate with GME data management systems (e.g., New Innovations, MedHub,) to develop guides for management and synthesis of all available data to inform and optimize visualizations and dashboards. Include Electronic Health Record Data so patient care processes and outcomes can be considered.^2934^
Data Analyses & Visualization	Identify patterns in the data before interpreting or acting upon it. Use accurate visualization tools and consistently match the data to decisions to be made. Implement evidence-based strategies for data visualization during CCC meetings [16].	ACGME should automate cloud-based visualization processes for programs’ Milestones data to reduce their data processing burden, and provide program comparisons to aggregated national Milestone data.
Interpretation & Decision-Making	Establish shared understanding of data interpretation (e.g., standard setting processes, concept mapping) before reviewing individual performance data. Focus on data as a whole before focusing on individual trainees. Enhance and promote the development of SMART (Specific, Measurable, Achievable, Relevant, Timely) [27,31] goals to inform residents’ plans for improvement, including metrics for achievement. Better inform GME program directors about Predictive Probability Value (PPV) data to inform CCC decisions and enhance outcomes for trainees and programs [24].	Capture data interpretations to continuously refine and test decision-making utility. Create robust educational databases to benchmark and integrate ACGME PPV data [24] to provide insights about curriculum and rotation structure, trainee trajectory of acquiring competence, and inform readiness for graduation and independent practice decisions [27].
Contributing to Generalizable Knowledge	Encourage scholarly activities using de-identified data to contribute to the science of CBME. Study the informatics of learning as its own sub-discipline.	Integrate national and local data to provide precise estimates of trainee competency and ‘readiness’ for competency-based time-variable GME [7,13,14]. Support networks of Data Teams/Education Scientists and CCC members to share expertise [27].

**Figure 1.**
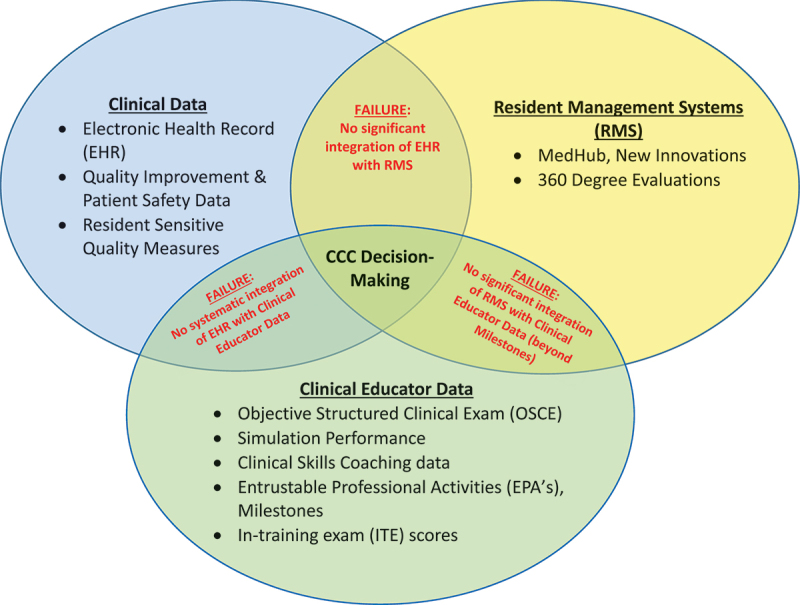
The Learning Analytics Landscape with Currently Existing Data Integration Failures.

Accreditation guides should point to strategies that can aid data analysis and decision-making [1,32]. Aspirational goals include collaborating with GME data management systems (e.g., New Innovations, MedHub) to develop step-by-step protocols for collating and synthesizing all available data to optimize data visualizations and dashboards. For example, programs can currently access the ACGME predictive probability values (PPVs), which indicate the probability of individual trainees reaching Milestone target Level 4 by graduation [21,24]. Figure 1 illustrates the GME LA landscape and calls out existing failures of data integration for CCCs [33]. Promising studies that link educational quality to patient data and outcomes, underscore the importance of data integration and analytics [19,34–37]. Integration of patient electronic health record data would complement trainee multi-source evaluation data, enhance the CCC decision-making process [34,37], and has been shown to enrich resident feedback and learning [38]. Lastly, creating formal networks of data teams, educational scientists, and CCC members to share complementary LA expertise and collaborate on scholarship would further develop this important work [19,23,27].

## Educator Development and Learning Analytics

Educators and trainees need to learn how to manage large streams of quantitative [39] and qualitative [40] data output as they flow to customized resident portfolios to inform CCC decisions and Individualized Learning Plans (ILPs) [28,41]. CCC members need a certain level of analytics literacy toward becoming ‘Diagnostic Assessors’ that address knowledge gaps [27]. Additional opportunities involve having data that informs both faculty and program development [12,42]. Table 2 outlines recommendations for both educator and program development to apply analytics to CCC processes. Rich LA can enhance trainee development and foster a growth mindset promoting self-reflection, self-directed learning, and co-production of ILPs [1,7,17,25,28,48–51].

**Table 2.** Effective Use of Learning Analytics by CCCs: Educator Development.

Essential Educator Development Areas for Learning Analytics	*Recommendations for Educator Development on the Use of Learning Analytics in CCCs*	
	Practical and Short-Term	Aspirational and Future-Oriented
Practical Applications of Learning Analytics	CCC members participate in ongoing professional development including: a) how evaluators contribute to improved data quality; b) practical data visualization and interpretation; and c) how to synthesize data into actionable feedback for trainees about what is needed to progress [34,38]. Educator development to ensure analysts, GME program leadership, CCC members, and coordinators understand how to interpret and integrate national PPV Milestone data with local program-level multi-source (360-degree) evaluation data [24,43]. Guidance on how to implement Milestones 2.0 recommendations [22]. Trainee development to foster a growth mindset, promote reflection & self-directed learning, and fully inform co-production of ILPs. Educators work together differently by imbedding ongoing innovative learning experiences into educational work routines. More fully develop raters across clinical rotations to improve completion and accuracy. Iteratively check in with CCC members periodically to increase faculty engagement and ownership over assessment/feedback processes.	Encourage faculty to undertake educational research training via MS, MEd, MHPE, MPH [44–46] to cultivate a pathway [27] for educational scientists and contribute scholarship to advance competency-based time-variable GME training [7,13,14]. Conduct research on how best to improve Learning Analytics and effective CCC processes to enhance competency-based medical education outcomes and competency-based time-variable GME training [13,14] National or regional networks are needed to study LA and CCC while sharing and pooling data and resources to move programs toward becoming master CBME educators and assessors.
Shared Mental Model of Learning Analytics	The Shared Mental Model section of the CCC Guidebook^1 p^ ^18^ should include an application of data generated by learning analytics and how these data can inform CCC Milestone determination of trainee progression and program improvement [34,37,38].	Development and refinement of organizational learning models to allow for growth and to take advantage of new technologies.
Educator Participation in Multidisciplinary Teams	ACGME should require that data teams be trained in data quality, management, and longitudinal processing. Programs should link to relevant hospital information systems and engage with learning analytics experts, and national datasets. Engage minoritized communities about collection of Diversity, Equity, Inclusion, and Social Justice data [47].	Establish training programs for physicians, as occurred with biomedical training programs [46], to cover costs of advanced educational science training. Fund programs offered through the ACGME and the National Library of Medicine to train Educational Informatics experts [44–46] Data team includes Masters or PhD trained educational scientists or physicians with informatics training with an in-depth understanding of GME program curriculum and competency-based multi-source assessments [40].

In terms of educator development, the 2021 JGME supplement [22] proposes that new CCC member orientation includes: 1) assessment and the use of the Milestones; 2) group decision-making; 3) awareness of biases; and 4) the impact of CCC decisions on patient care outcomes [12]. We propose that LA and data interpretation training be included in this orientation along with ongoing educator development that promotes a shared mental model of how informatics can support CCC processes and decisions [12,52]. In addition, simultaneously developing educators and trainees could be done by creatively imbedding ongoing innovative learning experiences on data quality and processes into current educational routines [12,53].

In the more distant future, GME would benefit from faculty trained in educational research through formal training and clinical informatics programs sponsored by the National Library of Medicine [44], ACGME [45], and/or biomedical programs for healthcare professionals [46]. This trained cadre could then ensure CCC members are fully informed about practical LA principles. Lastly, more research is needed to improve the utility of LA and effectiveness of CCCs long-term using well-established outcomes (e.g., tracking GME outcomes [23], board certification status, and disciplinary actions) [19,20,54].

## Next steps in supporting these considerable efforts

CCCs engage core GME faculty and program coordinators who balance multiple competing demands [55]. Ideally, a well-designed analytics infrastructure allows for the utilization of more data with less effort [32,56]. Investing in additional team members, ideally education scientists, can accelerate use of LA within CCCs [27]. Options for gaining additional resources include: 1) negotiating with the sponsoring institution to obtain funds or share analytic personnel/resources; 2) applying for local, regional, or national funding; 3) using departmental funds; or 4) altering ACGME requirements [57] to require this work.

Mechanisms for funding GME need to change to enable these kinds of innovations. Applying for grants to support this work as educational innovation is possible, but would not lead to a sustained infrastructure to support improvement [58]. The availability of departmental funds will likely vary by discipline, as those with high clinical revenue may be more likely to provide support. Similarly, large programs with research sections or university-based, affiliated or university administered programs may find expertise in informatics departments, schools of public health or education schools to guide integration of learning analytics into GME processes. Altering or adding program roles and full-time equivalent requirements in ACGME Common Program Requirements [57] may be complex due to employment laws, but times are changing, and residency training funding mechanisms also need revisions. A recent communication with Eric Holmboe, MD, Chief Research, Milestones Development and Evaluation Officer at the ACGME, elucidated that the ACGME is beginning a digital transformation and exploring learning analytics as part of that transformation (Cite: Personal Communication 1/26/23).

In summary, action is needed to fully realize CBME in residency training. Evolutionary pathways for inclusion of LA and educator and trainee development in guiding literature, such as the next CCC Guidebook [1] and ACGME Common Program Requirements [57], would help advance these efforts. Collaborating to co-produce generalizable LA processes with external stakeholders, including Residency Data Management Systems, to inform efficient CCC processes will be essential to advance CBME.
